# Current trends and perspectives of scoliosis research society travel fellows

**DOI:** 10.1007/s43390-024-00962-4

**Published:** 2024-09-20

**Authors:** Aditya Joshi, Arash Kamali, Jordan Helbing, Michelle C. Welborn, Steven W. Hwang, Amit Jain, Khaled Kebaish, Hamid Hassanzadeh

**Affiliations:** 1 Department of Orthopaedic Surgery, Johns Hopkins Hospital, Baltimore, MD USA; 2 Uniformed Services University School of Medicine, Bethesda, MD USA; 3 Department of Orthopaedic Surgery, Shriners Hospital for Children Portland, Portland, OR USA; 4 Department of Neurosurgery, Shriners Hospitals for Children-Philadelphia, Philadelphia, PA USA; 5 Present Address: Department of Orthopaedic Surgery, Johns Hopkins Medicine, National Capital Region, 6420 Rockledge Dr, Suite 2200, Bethesda, MD 20817 USA

**Keywords:** Scoliosis, Travel fellowship, Research, Education, Global

## Abstract

**Purpose:**

This study aims to measure the impact of the Scoliosis Research Society’s travel fellowship on a spinal surgeon’s career.

**Methods:**

A non-incentivized survey was sent to 78 previous SRS junior travel fellows from 1993 to 2021. The questionnaire assessed fellowship influence on academic and administrative positions, professional society memberships, and commercial relationships. The trend of these quantitative measures was created according to a compounded annual growth rate (CAGR) calculation of the reported values. The Scopus database was queried for all fellows’ publication counts and h-index before the fellowship, as well as 3 years, 5 years, and currently after the fellowship. A control cohort of matched surgeons who did not participate in travel fellowships was used to compare research productivity measures relative to travel fellows.

**Results:**

This study had a 73% response rate. Over the periods of 3–5 years after the fellowship, and up to the present, the mean publication count increased by 31.0%, 31.6%, and 46.4%, respectively. Over the same interval, the mean h-index increased by 19.5%, 17.3%, and 11.3%, respectively. From the year of their respective fellowship to present day, the fellows observed a mean CAGR of + 3.2% in academic positions, + 6.7% in administrative positions, + 2.3% in society memberships, and + 4.7% in commercial relations. Previous fellows concurred the fellowship changed their clinical practice (42.1% Strongly Agree, 36.8% Agree), expanded their network (71.9% Strong Agree, 24.6% Agree), expanded their research (33.3% Strongly Agree, 54.4% Agree), and improved their surgical technique (33.3% Strongly Agree, 49.1% Agree).

**Conclusion:**

Robust feedback from previous fellows suggests a traveling fellowship has a meaningful impact on a surgeon’s research productivity and career achievements.

**Supplementary Information:**

The online version contains supplementary material available at 10.1007/s43390-024-00962-4.

## Introduction

Following the pandemic, medical education shifted toward digital platforms [1]. This transition to web-based training profoundly impacted surgical trainees who depend on clinical exposure and surgical case volume to successfully progress in their training [2]. A growing body of evidence has commended the use of surgical simulations to achieve milestones in orthopedic training [3]. However, these adaptations are not without limitations, and there is growing interest in returning to traditional practices such as travel fellowships [4]. Traveling fellowships have emerged as a cornerstone for surgeons seeking to broaden their horizons in clinical skills, research, and leadership within the global healthcare landscape. These fellowships, which range from several weeks to a few months, immerse participants in diverse healthcare environments, offering them a unique platform for professional development, knowledge exchange, and networking [5]. Surgeons have the opportunity to learn from renowned experts, absorb advanced surgical techniques not readily available in their home countries, and significantly enhance their clinical practice and patient care upon their return.

Among these transformative programs, the Scoliosis Research Society (SRS) Traveling Fellowship exemplifies a specialized focus on advancing knowledge and techniques in spinal deformities. This prestigious fellowship enables early to mid-career spine surgeons to visit leading spine surgery centers worldwide, fostering an environment for professional growth, surgical skill enhancement, and the initiation of research collaborations. Participants in the SRS Traveling Fellowship engage deeply with the latest advancements in spinal deformity surgery, benefiting from the mentorship of world-renowned experts and the opportunity to share their own insights and experiences [6].

Participation in traveling fellowships notably affects research productivity, with surgeons demonstrating a significant increase in publications, especially in high-impact journals [7]. This trend contributes to the individual’s career growth and propels the entire orthopedic field forward, showcasing the essential role of fellowships in fostering a deep engagement with academic research. These programs further enhance professional networking and collaboration, allowing fellows to build lasting relationships with prominent figures in the field worldwide [8]. Such connections often result in joint research projects, publications, and participation in international conferences, thereby enriching the surgeon’s academic and clinical practice and positioning them for leadership roles in academia, clinical settings, and healthcare policy [9]. The purpose of this study was to qualitatively and quantitatively assess the impact of the SRS traveling fellowship on an emerging spine surgeon’s career.

## Methods

### Sampling method

A list of previous awardees for the SRS traveling fellowship is publicly available on the SRS Past Award Winners webpage [6]. To focus on the effects of the fellowship on surgeons early in their career, only junior fellows were examined for this study. The SRS webpage defined junior fellow as SRS Active and Candidate members in good standing who are under age 45 and earlier in their career at the time of application. Senior fellows were defined as those who were senior members of SRS, more advanced in their career, and made significant contributions to spinal deformity surgery. Correspondence emails for each respective fellow were queried from the PubMed database. A non-incentivized anonymous survey email was sent to 78 junior fellows listed from 1993 to 2021. The questionnaire is included in the supplementary material. A control cohort was defined by querying each fellow’s academic institution during the year of their fellowship and matching a colleague surgeon who did not participate in a travel fellowship.

### Research productivity measures

Using the publicly available Scopus database, the h-index and publication count of all 78 fellows prior to their fellowship year, exclusive, were recorded. Additionally, the h-index and publication count were also curated from the database for 3–5 years after the completion of their fellowship, including those years. Current h-index and total publications were also compiled. These measures were also accrued for the control surgeons.

### Statistical analyses

Descriptive statistics were used to organize research productivity measures and survey responses. Mean h-index, number of publications, academic positions, administrative positions, society memberships, and commercial relations were recorded. Percent change in h-index and publication count following fellowship year were calculated. A Student’s t test was used to determine any differences in research productivity between the travel fellows and control cohort. Similarly, a paired t test was used to assess for any differences among cohorts in the subcohort analyses. A logistic regression model was used to determine the independent effect of travel fellow demographics on academic success following the fellowship. In order to adjust for years since fellowship, the number of academic positions, administrative positions, society memberships, and commercial relations following the fellowship was assessed by the following formula: Compounded annual growth rate = (Y2/Y1)^1/(Y2−Y1)^−1. The compounded annual growth rate (CAGR) is a reliable metric for assessing annual changes, frequently employed in trend analysis. Its effectiveness lies in its capacity to mitigate the influence of short-term fluctuations on long-term trends [10]. Trend analysis was considered statistically significant at *p* < 0.05. All statistical analyses were performed on R Studio (version 4.3.1).

## Results

### Travel fellow characteristics

The study had a 73% (*n* = 57) response rate. 49% (*n* = 28) of respondents were North American and 51% (*n* = 29) international. The mean number of years since their fellowship was 13.2. Mean publication count before and after the fellowship was 54.1–202.2, respectively. Mean h-index before and after the fellowship was 19.2–34.4, respectively (Table 1).

**Table 1:** Characteristics of fellows who responded to online survey

Fellow characteristics	
North american (%)	28 (49%)
International (%)	29 (51%)
Mean years since fellowship	13.2
Mean publication count before fellowship	54.1
Mean publication count after fellowship	202.2
Mean annual percent change of publication	47.7%
Mean h-index before fellowship	19.2
Mean h-index after fellowship	34.4

### Research achievements

For all fellows examined in this study, the mean publication count and h-index had annually increased by 31.0–19.5%, respectively, 3 years after the fellowship. At the 5 year mark, the mean publication count and h-index had annually increased by 31.6% and 17.3%, respectively. Present day publication count and h-index had increased by 46.4% and 11.3%, respectively. In contrast, the control cohort observed a mean annual percentage increase in publications by 25.6%, 19.7%, and 40.3% at the 3 year, 5 year, and present day time periods, respectively. Regarding h-index, the control cohort had a 7.7%, 14.5%, and 10.5% increase at the 3 year, 5 year, and present day time periods, respectively (Table 2).

**Table 2:** Research productivity across all 78 listed fellows and control cohort

Research characteristic	Travel fellows	Control surgeons	*p* value
Publications after 3 years	31.0%	25.6%	0.036
Publications after 5 years	31.6%	19.7%	< 0.001
Publications present day	46.4%	40.3%	0.072
h-index after 3 years	19.5%	7.7%	0.048
h-index after 5 years	17.3%	14.5%	0.32
h-index present day	11.3%	10.5%	0.67

Reported percentages are the mean annual change for the listed time period. *p* values report statistical significance of the simple *t*-test used to compare the two cohorts

### Career achievements

Fellows had a 40.4% increase in academic positions (CAGR: + 3.2%, *p* = 0.012), 179% increase in administrative positions (CAGR: + 6.7%, *p* = 0.004), 37.5% increase in society memberships (CAGR: + 2.3%, *p* = 0.036), and 86% increase in commercial relationships (CAGR: + 4.7%, *p* = 0.011). Trend analysis demonstrated a significant increase in all recorded career achievements following the fellowship (*p* < 0.05) (Table 3).

**Table 3:** Number of career milestones before and after fellowship year

	Before fellowship	After fellowship	CAGR	p value
Academic Positions	2.0	2.9	+ 3.2%	0.012
Administrative Positions	0.9	2.7	+ 6.7%	0.004
Society Memberships	4.0	5.5	+ 2.3%	0.036
Commercial Relationships	1.8	3.3	+ 4.7%	0.011

Compound annual growth rate (CAGR) analysis accounts for years since fellowship to determine if significant trend is present (*p* < 0.05)

### Fellow perspectives of the fellowship

**Fig. 1:**
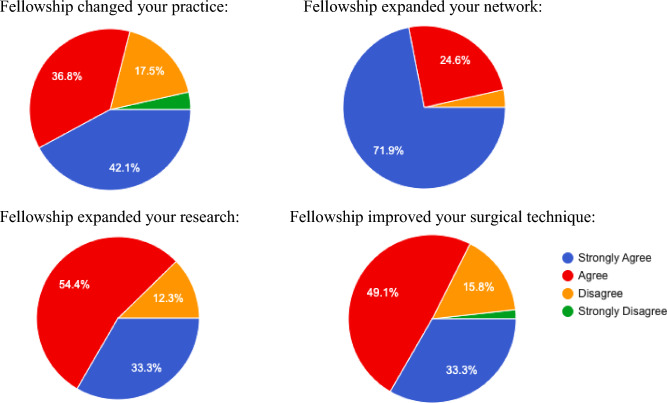
Fellow opinions regarding travel fellowship influence in various aspects of their career

In their qualitative responses, previous fellows noted the fellowship changed their clinical practice (42.1% Strongly Agree, 36.8% Agree, 17.5% Disagree, 3.5% Strongly Disagree), expanded their network (71.9% Strong Agree, 24.6% Agree, 3.5% Disagree), expanded their research (33.3% Strongly Agree, 54.4% Agree, 12.3% Disagree), and improved their surgical technique (33.3% Strongly Agree, 49.1% Agree, 15.8% Disagree, 1.8% Strongly Disagree). (Fig. 1).

### Subcohort analyses

After stratifying according to region, the average North American fellow had a statistically significant increase in mean publication count since the year of fellowship relative to their international counterparts (57.6% vs 31.1%, *p* value = 0.04). There were no other statistically significant differences in research productivity among the two cohorts (Table 4).

**Table 4:** Research productivity across all listed fellows according to region

Research characteristic	North american fellows	International fellows	*p* value
Publications after 3 years	34.0%	28.1%	0.43
Publications after 5 years	35.7%	26.3%	0.27
Publications present day	57.6%	31.1%	0.04
h-index after 3 years	22.0%	16.0%	0.45
h-index after 5 years	19.1%	15.1%	0.54
h-index present day	14.3%	7.6%	0.29

Reported percentages are the mean annual change for the listed time period. *p* values report statistical significance of the simple *t*-test used to compare the two cohorts

The fellows from 2007 to 2021 were stratified by travel regions (*n* = 37). The fellows who traveled to Europe (*n* = 7) had greater odds of academic benefit (OR 1.34, 95% CI 1.11–1.57; *p* = 0.024) compared to fellows who traveled to the USA (*n* = 16). There was no statistically significant difference among fellows who traveled to Asia (*n* = 14) (OR 0.77, 95% CI 0.22–1.32; *p* = 0.978) compared to the USA (Table 5). The percent change in publication count at 3 years, 5 years, and present was also greatest among the fellows who traveled to Europe at 26.9%, 26.8%, and 33.9% respectively. The h-index also changed greatest among fellows who traveled to Europe at the 3 years, 5 years, and present, with 13.2%, 9.0%, and 5.8% respectively. See Table 6 for the complete percent change of publications and h-index by travel region.

**Table 5:** Odds ratios associated with significant academic benefits

Fellow characteristic	Academic benefits odds ratio (95% CI)	*p* value
Year of Fellowship (ref: 1993–2002)		
2003–2012	1.67(0.37–3.07)	0.533
2013–2023	9.97(2.12–17.82)	0.011
Region of origin (ref: N. America vs. international)	0.73(0.31–1.15)	0.649
Region traveled to in fellowship (ref: USA)		
Asia	0.77(0.22–1.32)	0.978
Europe	1.34(1.11–1.57)	0.024
Strong academic background	0.89(0.25–1.52)	0.789

Significant academic benefits was defined as any fellow who achieved higher than the mean annual percentages listed in Table 1. Strong academic background was defined as any fellow with a publication count 100 + or h-index 25 +

**Table 6:** Mean percentage change in publication count and h-index by region of travel at 3 and 5 years post fellowship and present day

Publication count % change	3 year mean	5 year mean	Present mean
Asia (*n* = 14)	18.4%	23.5%	25.2%
Europe (*n* = 7)	26.9%	26.8%	33.9%
USA (*n* = 16)	22.2%	19.8%	21.4%

H index % change			
Asia (*n* = 14)	8.5%	8.2%	4.3%
Europe (*n* = 7)	13.2%	9.0%	5.8%
USA (*n* = 16)	9.8%	7.9%	5.3%

Limited to travel fellows from 2007 to 2021 due to the obtained list of travel regions

When fellows were analyzed in subcohorts of year of fellowship, fellows who participated from 2013 to 2023 were found to have significantly increased odds of academic benefits (OR 9.97, 95% CI 2.12–17.82, *p* = 0.011) compared to the 1993–2002 cohort, while the 2003–2012 cohort was not statistically significant. There were no statistically significant differences in academic benefit based on region (Table 5).

We considered fellows who had a publication count greater than 100 or an h-index greater than 25 prior to the travel fellowship as having a strong academic background. Among these fellows, there was no difference in association with academic benefit (OR 0.89, 95% CI 0.25–1.52; *p* = 0.789) (Table 5).

We defined fellows to have significantly benefited academically from the fellowship by those who achieved higher than the mean annual percent change of publications (47.7%, Table 1). In total, there were 11 fellows deemed to have significantly benefitted from the fellowship academically. Among these fellows, they had an average of 14.7 publications prior to the fellowship, with an average annual increase by 78.9% at the 3 year mark, 72.6% at the 5 year mark, and 153.1% at present day.

## Discussion

The evolution of traveling fellowships since World War II reflects a shift from mere technical skill acquisition to a more holistic approach that includes leadership development and cultural exchange [11]. This transformation underscores the multifaceted impact of these fellowships on an orthopedic surgeon’s career, highlighting not only the advancement of clinical expertise but also the enhancement of research productivity and the establishment of a robust professional network [7]. As graduate medical education returns to in-person opportunities, traveling fellowships should be recommended as a means to advance clinical training and promote cultural diversity. The SRS traveling fellowship demonstrates the value of such specialized programs in shaping the future of orthopedic surgery.

Results of this study suggest that the SRS traveling fellowship plays a transformative role in a spine surgeon’s career. The fellows observed experienced the greatest increase in research productivity within the first three years of their fellowship, continuing up to the five-year mark. This surge in publication count and h-index tapered off by the present day. While we found fellows who participated from 2013 to 2023 to have the greatest odds of academic benefit, it is important to note the transformation in medical publishing from printed journals to digital publications allowing for greater opportunities and possibly contributing to the increase seen in this cohort. Acevedo et al. found no significant differences in research productivity among various orthopedic residency programs. However, they noted considerable improvements in research output after the completion of residency. Specifically, highly motivated surgeons who pursued careers in academic medicine showed the most significant increase in research productivity [12]. A plausible explanation for the research trend observed in this study is the selection of highly motivated surgeons for travel fellowships due to the role these programs play in their career trajectories. Individual reports from prior SRS traveling fellowship cohorts further endorse this perception of the fellowship [13–15].

Despite the majority positive responses from fellows regarding the fellowship’s impact on clinical practice, research, network, and surgical technique, several respondents reported no such benefit in one or multiple of these facets. Due to the survey nature of this study and the limitations due to these categories being broad, specific factors potentially contributing to the lack of benefit reported by fellows cannot be definitively assessed. However, it is important for future studies to evaluate potential factors to best improve the fellowship for future participants.

Limitations include the survey nature of this study. The majority of respondents are likely to comment positively on the influence of the fellowship. Qualitative responses from this study demonstrated that the travel fellowship shaped many of the clinical and surgical practices later adopted by its participants. However, we also noted several responses critiquing the influence of the traveling fellowship on different aspects of their careers, suggesting that response bias may have been limited (Fig. 1). Previously, Hollyer et al. found that actual practice changes four years after a travel fellowship were lower than initially noted [16]. Travel fellowships are generally designed for surgeons in the early to middle stages of their careers. While the survey respondents reported they strongly agreed or agreed that the fellowship changed their clinical practice, the question was left broad limiting the conclusions we could draw. Therefore, it is important to study the long-term impact these fellowships have on their surgical practices. We also recognize that changes in the number of career milestones, such as academic positions and society memberships, cannot be solely attributed to the fellowship. Therefore, in this study, we compared percentages of career achievements with the CAGR analysis and believe this provides valuable insight into the observed changes. Future studies should include a controlled trial with matched surgeons who do not participate in a traveling fellowship to isolate its effects.

The experiences from a traveling fellowship not only foster personal and professional growth but also have the potential to shape the future of orthopedic surgery through the dissemination of knowledge and innovation. As the healthcare landscape continues to evolve, the role of traveling fellowships in preparing surgeons for leadership roles within this dynamic environment cannot be overstated. The importance of such fellowships is increasingly recognized, warranting a need for scholarly publications exploring their benefits. This academic interest is paralleled by a critical examination of fellowship selection processes and the optimal use of digital platforms to inform and attract candidates. Despite technological advancements, the variability in how fellowship programs present themselves online suggests a need for improvement [17], emphasizing the role of effective communication in guiding prospective applicants through their decision-making processes [18]. Likewise, the availability of such pivotal opportunities needs to increase as well.

## Supplementary Information

Below is the link to the electronic supplementary material.

Supplementary file1 (DOCX 14 KB)

## Data Availability

Raw data from survey is not publicly available to preserve individuals’ privacy under Institutional Review Board guidelines.
