# Supplementary material for: Current trends and perspectives of scoliosis research society travel fellows
Source: Spine Deform. 2024 Sep 20;13(1):65–71. doi: 10.1007/s43390-024-00962-4 (PMC11729127; doi:10.1007/s43390-024-00962-4)
Supplement: Supplementary file 1 — Supplementary file1 (DOCX 14 KB) [file 43390_2024_962_MOESM1_ESM.docx]

1. Number of academic positions (i.e promotion in professorship) **BEFORE** the fellowship:
2. Number of academic positions **AFTER** the fellowship
3. Number of administrative positions (i.e. promotion to department chair) **BEFORE** the fellowship
4. Number of administrative positions **AFTER** the fellowship
5. Number of society memberships **BEFORE** the fellowship
6. Number of society memberships **AFTER** the fellowship
7. Number of commercial relations 
   (i.e consultant position) **BEFORE** the travel fellowship
8. Number of commercial relations **AFTER** the travel fellowship
9. Did the fellowship change your clinical practice?
   1. Strongly Agree
   2. Agree
   3. Disagree
   4. Strongly Disagree
10. Did the fellowship expand your network?
    1. Strongly Agree
    2. Agree
    3. Disagree
    4. Strongly Disagree
11. Did the fellowship expand your research?
    1. Strongly Agree
    2. Agree
    3. Disagree
    4. Strongly Disagree
12. Did the fellowship improve your surgical technique?
    1. Strongly Agree
    2. Agree
    3. Disagree
    4. Strongly Disagree
